# The Book World of Medicine and Science

**Published:** 1903-09-12

**Authors:** 


### The Book World of Medicine and Science.

Lessons in Disinfection and Sterilisation. By F. W. Andrewes, M.D., F.R.C.P., D.P.H., Lecturer on Pathology, Pathologist and Sanitary Officer to St. Bartholomew's Hospital, London. (London: J. and A. Churchill. Pp. 222. Illustrations 31. 1903. Price 3s. net.)

This is an eminently practical book. The very foundation of surgery and much of the good work of medicine are intimately associated with a knowledge of bacteria, their life-history and effects. To the nurse, to the medical student, to the practitioner, and even to the layman there is in this short work so much that is not only interesting, but needful, that we do not hesitate to recommend it as heartily as does Dr. Klein in the letter which prefaces it. The book is divided into ten parts, each being practically a complete lesson in itself. After a careful and clear survey of the nature, growth, and chemical activities of bacteria the author proceeds to the discussion in more detail of the methods of disinfection. Sections are introduced which describe the means of disinfection by heat, by chemicals, through the air, and by filtration. A very important chapter is that dealing with disinfection as applied in surgery and midwifery; and another, that relating to disinfection in medical cases. The last lesson is concerned with a short account of the chief bacteria found in the more important specific diseases. The book terminates with a graphic account of various practical exercises and demonstrations whereby the learner may prove much for himself by actual experiment. An example of one of these may be given. It is an experiment to test the efficiency of steam disinfection:—"Take an old directory or other book of no value—the bigger and thicker the better. Open it in the middle, and with a penknife cut a small cell in the central pages. In this cell place some fragments of paper smeared with a sporing culture of the hay bacillus. . . . Close the book, and make it up into a parcel with brown paper and string. Wrap it up in old cloths, blankets, or other articles until the parcel is of considerable bulk, and again finish with brown paper and string. Then send it to the steam disinfector to be passed through with other goods. . . . Open the book cautiously, and with sterilised forceps transfer the spore-infected fragments of paper to the broth tubes. . . . Incubate. . . . They should remain perfectly sterile. . . ." Surely this is a severe, and yet wholly legitimate test!

La Méthode de Prokhorow dans le Traitement de la Syphilis. Par François L. Nario. (Buenos Aires: Imprimerie Zenorio.)

In this little publication the author sets forth the advantages of intra-muscular injections of mercury in the treatment of syphilis, and brings forward a large number of cases which he has had under his care, and which he believes go to prove that this method of administering mercury is the one to be adopted by choice. Briefly stated, Prokhorow's method consists of injecting large doses of biniodide of mercury into the muscles of the buttock. The amount that Prokhorow himself recommended and made use of was three milligrammes per kilo of the patient's weight for an adult, and half this quantity for infants. The actual solution used was made up as follows:—Biniodide of mercury 0·30 grammes, iodide of potassium 0·60 grammes, distilled water 100 c.c. According to the number of kilos that the patient weighed, the number of c.c. of the solution to be injected was regulated. That patients can tolerate these very large doses depends, according to Dr. Nario, on two things. In the first place elimination takes place very rapidly; and secondly a double iodide of mercury and potassium is formed which is not nearly so toxic as the simple biniodide of mercury. Owing to the large bulk that must be injected into an adult if Prokhorow's solution be used, Dr. Nario recommends more concentrated solutions, using only 25 c.c. of water instead of 100 c.c. In making the injection strict antiseptic precautions are observed, the skin of the buttock and everything used during the procedure being carefully sterilised. In one case, where the injection was made subcutaneously, a small patch of gangrene occurred round the site of puncture. No such accident occurred in any of the cases where the needle was introduced more deeply. The only drawbacks to the method employed have been local pain and swelling, and it was found that these could be to a great extent avoided by giving the smaller and more concentrated injections, and by keeping the patient quiet for some time after the operation. No case of stomatitis, gastralgia, dyspepsia, diarrhœa, or nephritis was observed to follow. It is to be noticed that Dr. Nario refers to nephritis as if in his experience it were a frequent complication of mercurial treatment by inunction or by oral administration. In adults he followed Prokhorow's plan of repeating the injections every six days, but in infants this interval was found to be too short. He considers that when five such injections have been made a long interval should be allowed to pass before the treatment is repeated. In the case of infants a gain in weight is taken to be the most reliable indication of progress. If the symptoms of syphilis have disappeared, but the child does not increase in weight, either the syphilitic cachexia is advancing or the mercury is debilitating the patient. In the former case, injections should be repeated every fifteenth day, in the latter every thirtieth. Usually all secondaries disappear after three administrations. The booklet is more especially interesting from the fact that Dr. Nario has had exceptional opportunities for studying syphilis, chiefly among children who have inherited the disease.

Handbook of Obstetric Nursing. By F. Haultain, M.D., and J. H. Ferguson, M.D. Fourth Edition. Revised and enlarged. (Edinburgh and London: Young J. Pentland. 1902. Crown 8vo. 264 pages. 5s.

A Short Practice of Midwifery for Nurses. By Henry Jellett, M.D. (London: J. A. Churchill. Crown 8vo. 336 pages. 6s.)

These two little books are sufficiently described by their title. The information contained in them is accurate and clearly expressed. They are both well written and well planned. We would advise any nurse who reads the first to also read the second, and we trust that every midwife who reads the second will make herself *au fait* with the details of the first. We commend both books heartily.

